# Probing a Polar Cluster in the Retinal Binding Pocket of Bacteriorhodopsin by a Chemical Design Approach

**DOI:** 10.1371/journal.pone.0042447

**Published:** 2012-08-03

**Authors:** Rosana Simón-Vázquez, Marta Domínguez, Víctor A. Lórenz-Fonfría, Susana Álvarez, José-Luís Bourdelande, Ángel R. de Lera, Esteve Padrós, Alex Perálvarez-Marín

**Affiliations:** 1 Departament de Bioquímica i de Biologia Molecular and Centre d’Estudis en Biofísica, Universitat Autònoma de Barcelona, Barcelona, Spain; 2 Departamento de Química Orgánica. Facultad de Química, Universidade de Vigo, Vigo, Spain; 3 Departamento de Química (Química Orgánica) Universitat Autònoma de Barcelona, Barcelona, Spain; University of Lethbridge, Canada

## Abstract

Bacteriorhodopsin has a polar cluster of amino acids surrounding the retinal molecule, which is responsible for light harvesting to fuel proton pumping. From our previous studies, we have shown that threonine 90 is the pivotal amino acid in this polar cluster, both functionally and structurally. In an attempt to perform a phenotype rescue, we have chemically designed a retinal analogue molecule to compensate the drastic effects of the T90A mutation in bacteriorhodopsin. This analogue substitutes the methyl group at position C_13_ of the retinal hydrocarbon chain by and ethyl group (20-methyl retinal). We have analyzed the effect of reconstituting the wild-type and the T90A mutant apoproteins with all-*trans*-retinal and its 20-methyl derivative (hereafter, 13-ethyl retinal). Biophysical characterization indicates that recovering the steric interaction between the residue 90 and retinal, eases the accommodation of the chromophore, however it is not enough for a complete phenotype rescue. The characterization of these chemically engineered chromoproteins provides further insight into the role of the hydrogen bond network and the steric interactions involving the retinal binding pocket in bacteriorhodopsin and other microbial sensory rhodopsins.

## Introduction

**Figure 1:**
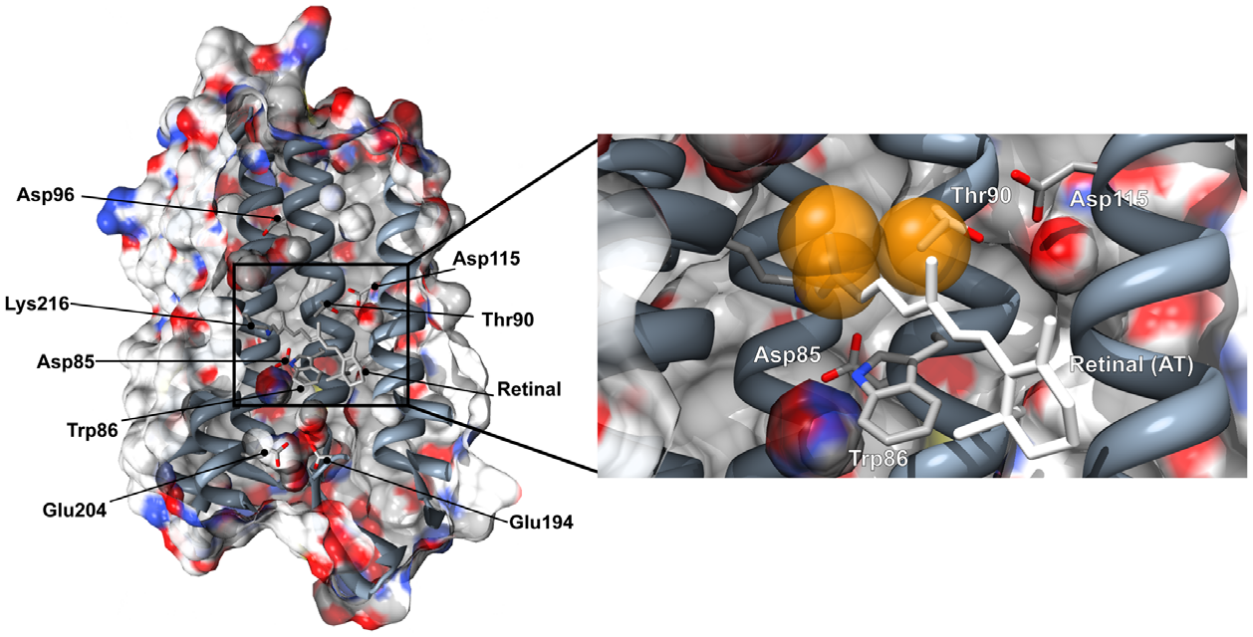
bR tridimensional structure. **A**. Surface and cartoon representation of bR (pdb code 1c3w) indicating the most relevant residues for this study and for proton transport. Surface coloring is coded by element (C, white; O, red: N, blue; S, yellow). In the expanded view, the interacting methyl groups are indicated in gold color.

Retinal-related proteins are chromoproteins represented among the Archaea, Eubacteria and Eukarya domains, working either as sensors or as ion pumps [1]. These membrane proteins do not only share the binding of a retinal chromophore through a protonated Schiff Base (SB), but they also share a common fold of seven transmembrane helices as revealed by currently available X-ray crystallographic structures [2], [3], [4], [5], [6]. Bacteriorhodopsin (bR) from *Halobacterium salinarum* is a protein that converts light energy absorbed by a retinal molecule (covalently linked through a protonated SB to Lys216, Figure 1) into a proton electrochemical gradient across the membrane [7], [8], [9]. The disposition in the membrane of *H. salinarum* consists of a honeycomb lattice of bR trimers forming a natural 2D crystal, known as the purple membrane (PM) [10], [11], [12].

Bacteriorhodopsin presents two different ground state forms, known as the dark (DA) and light-adapted (LA) states. In the LA state the retinal adopts an all*-trans* configuration, with a negligible fraction of other retinal isomers [13]. Under this condition the absorption of light induces the ultrafast retinal isomerization to 13-*cis*, followed by a thermal relaxation process involving changes in the retinal and in the protein conformation known as the photocycle, resulting in the net transfer of one proton from the cytoplasm to the extracellular side of the membrane. The photocycle ends with the recovery of the ground state conformation containing all-*trans* retinal in less than 30 ms at room temperature [14].

In the dark the LA state relaxes thermally in around 20 minutes at 35°C to the most stable DA state, [15] consisting of an equilibrium mixture of 13-*cis* and all-*trans* retinal in an 2∶1 ratio. The photocycle of 13-*cis-*retinal involves the isomerization to all-*trans*-retinal, which relaxes unproductively within few milliseconds, eventually leading to the complete light adaptation of bR. Mutation of residues in the retinal binding pocket (RBP) or the use of artificial retinal analogues can alter the retinal isomeric composition in the DA and LA forms of bR as well as the rate of dark adaptation [16], [17], [18].

Some of our previous studies have shown that Thr90, a residue not directly involved in the proton-pump photocycle, is important to preserve the structure, the proton-pump activity, and the normal photocycle of bR [19], [20]. The Thr90 hydroxyl group is involved in an inter-helical hydrogen bond with Asp115 side chain and an intra-helical hydrogen bond with Trp86. It is also in steric contact with the retinal molecule, where the methyl group of Thr90 side chain contacts with the retinal hydrocarbon chain, specifically with the C_13_-methyl group of the retinal (Figure 1). As deduced from the D115A mutant, the Thr90-Asp115 interaction plays no evident structural role [21] even though it contributes to the thermal stability of bR [20]. The loss of the Thr90-Asp115 interaction in the D115A mutant has no detectable effect in the retinal isomeric composition of the LA and DA forms [22]; moreover, the architecture of the RBP is not altered [21]. The mutant D115A displays nevertheless an altered behavior on its photocycle, highlighting the functional/dynamical role of the inter-helical Thr90-Asp115 interaction [22].

The T90A mutation abolishes simultaneously all three mentioned interactions of Thr90 with Asp115, Trp 86 and the C_13_-methyl retinal, with relevant effects on the structure and photocycle of bR and in the paracrystalline arrangement of the PM. Mutation of Thr90 to Ala also affects dramatically the light to dark adaptation process [19], [20]. Interestingly, T90A has also been reported to be unstable for crystallization [21]. The mutant T90V, designed to maintain the interaction with the C_13_-methyl while losing those with Asp115 and Trp86, partially rescued some of the alterations observed in T90A [20].

Here, to further assess the structural and functional importance of the interaction of the retinal C_13_-methyl with Thr90 we have substituted the endogenous retinal by the analogue 13-ethyl retinal. Thus, the apoprotein form (bacterioopsin, bOp) of WT and T90A was reconstituted with either all*-trans* retinal (AT) or the analogue 13-ethyl retinal in *trans* configuration (13E-RET). Then, we characterized by diverse biophysical methods the behavior of these four forms in order to examine whether the extra group on C_13_, which should facilitate its interaction with Ala90, could restore some properties of WT in the T90A mutant.

## Materials and Methods

### Synthesis of Trans-13-ethylretinal (13E-RET)

**Figure 2:**
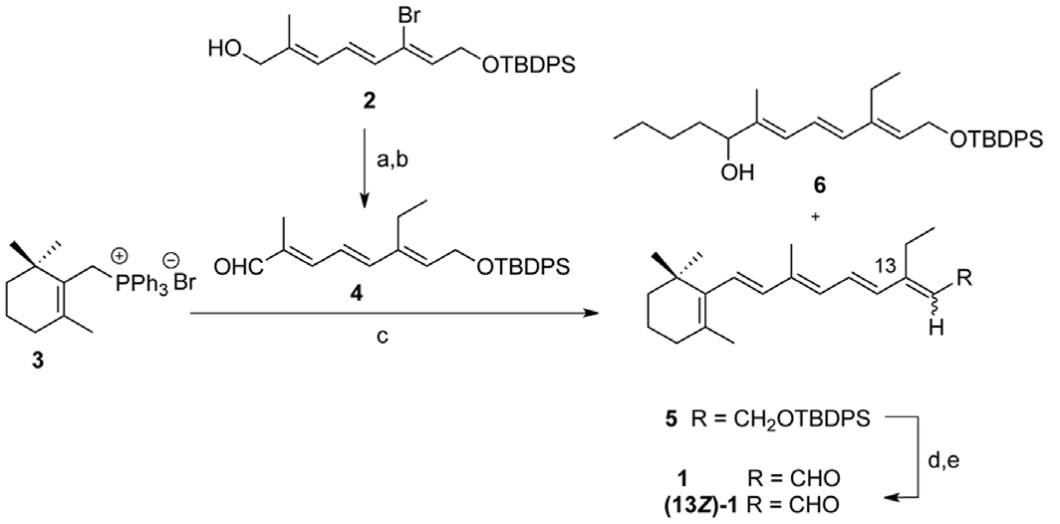
Synthesis overview. a) EtMgBr, NiCl_2_(dppp), THF, 0 to 40°C, 66%; b) MnO_2_, CH_2_Cl_2_, 25°C, 79%; c) *n*-BuLi, THF, aldehyde **4**, −78 to 0°C, (**6**, 70%; **7**, 16%); d) *n*-Bu_4_NF, THF, 25°C; e) MnO_2_, Na_2_CO_3_, CH_2_Cl_2_, 25°C, 60% both steps.

For the stereoselective synthesis of *trans*-13-ethylretinal **1**, we selected the Wittig reaction for the construction of the C7–C8 bond (Figure 2). The introduction of the ethyl group at position C_13_ was achieved by the Kumada-Tamao-Corriu cross coupling reaction catalized by Ni [23], [24]. Reaction between the already described bromide **2** [25] and ethyl magnesium bromide and subsequent oxidation of the alcohol obtained under basic conditions afforded trienal **4** in good yield. Wittig olefination of this aldehyde with the already described phosphonium salt **3** [26] afforded with complete selectivity the protected alcohol **5** in 70% yield; the product of the addition of base (*n*-BuLi) to the aldehyde **4** was also detected. Finally, deprotection of **5** with *n*-Bu_4_NF and rapid oxidation of the alcohol **6** with MnO_2_ under basic conditions did not prevent partial isomerization and afforded 13-ethylretinal **1** as a mixture of 13*E*/13*Z* isomers in a 2∶1 ratio, which were separated by HPLC (Preparative Nova Pak^®^ HR silica, 60 Å, 19×300 mm, 95∶5 hexane/EtOAc as eluent). Further information for the synthesis is provided in Methods S1.

### Expression and Photobleaching of the Purple Membrane

Purple membranes from T90A and from WT bR were expressed and purified from *H. salinarum* as described [27]. For retinal SB hydrolysis and removal from the RBP (protein bleaching), a stirred aqueous suspension of 10 mL containing 7 mg of PM was reacted with 1 M hydroxylamine hydrochloride at pH 7.5 and room temperature under continuous illumination (800 lux) for 3 hours. The bleached protein was centrifuged and washed three times in phosphate buffer (50 mM, pH 6.5) to remove the free retinal-oxime in the solution, and it was finally resuspended in 3 mL of the same buffer. A small fraction of the hydrolyzed retinal-oxime remained in the RBP or interacting with the bOp even after extensive washing, as evidenced by an UV-Vis broad band around 350 nm characteristic of retinal-oxime [27].

### Reconstitution of bOps with All-trans and 13-ethyl retinal

Suspensions of bOp (1.0×10^−5^ M) in phosphate buffer (50 mM, pH 6.5) were incubated with 1.5 equivalents of AT or the analogue 13E-RET dissolved in ethanol as described [28]. The rate of formation of the pigment with AT or 13E-RET was determined from the absorbance increase of the reconstituted pigment at its maximum absorbance wavelength, at around 570 nm.

### Asp85 pKa of the Retinal-reconstituted bOps

Retinal-reconstituted bOps in non-buffered conditions were microtitrated in the pH range from 7.5 to 2 by addition of small volumes of HCl or NaOH 0.1 M. After every pH change an UV-vis spectra was recorded. Protonation of the Asp85 causes a red-shift in the retinal absorbance, the so-called purple-to-blue form transition [29]. The corresponding absorbance changes at 615 nm as a function of pH were used to monitor this transition, and to determine the pK_a_ of Asp85 fitting the Henderson-Hasselbach equation to the data.

### Schiff Base Hydrolysis and Retinal Release by Hydroxylamine Hydrochloride

Suspensions of 1.0×10^−5^ M reconstituted bOp with AT or 13E-RET were treated with 40 mM hydroxylamine hydrochloride at pH 7.5 in buffer NaPi 5 mM in the dark. UV-Vis spectra were taken at different time intervals to follow the kinetics of the SB hydrolysis. The decay with time of the absorbance changes at the maximum wavelength (λ_max_) of the pigments followed a monoexponential equation, and its half-lifetime constant t_1/2_ was estimated.

### Thermal Stability

Analysis of thermal stability of dark-adapted samples at 0.75×10^−5^ M in phosphate buffer was carried out by recording absorption spectra in the UV-visible range as a function of temperature as described earlier [30]. The absorbance changes at the λ_max_ of the pigment band in the visible region *vs.* temperature were fitted to a sigmoidal equation yielding a T_m_.

### Photocycle Characterization of Retinal-reconstituted bOps by Flash Photolysis UV-Vis Spectroscopy

Flash-induced transient absorbance changes were monitored as described [20]. Photocycle reactions of purple membrane suspensions of retinal-reconstituted bOps at pH 7.0 in 3 mM sodium phosphate buffer and 150 mM KCl were followed by the transient absorbance changes at 410 nm for M intermediate, at 570 nm for the BR state, and at 660 nm for O intermediate as a function of time. Transient pH changes associated with the proton uptake and release in the bR photocycle were monitored using the pH probe pyranine. Flash-induced transient absorbance changes of protein suspensions in 150 mM KCl at pH 7.2 were measured in the presence of 50 µM pyranine at 460 nm, subtracting the response of the sample in the absence of the pH dye. The absorbance curves were normalized according to the apoprotein concentration determined by means of the absorbance at 280 nm in the UV spectrum.

## Results

### Incorporation of Retinal and Light-dark Adaptation

**Figure 3:**
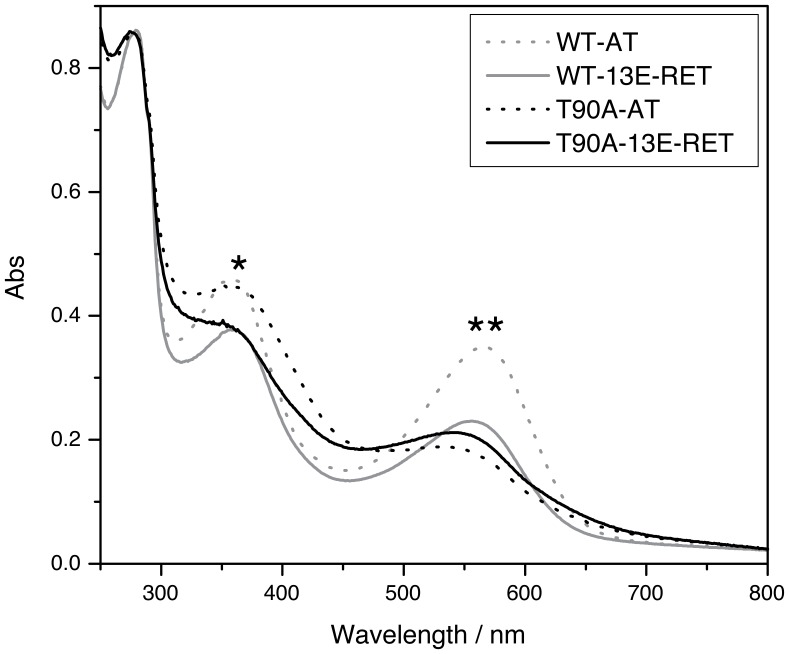
Retinal reconstitution assay. Spectra of the UV-Vis absorbance of the WT and T90A bOp after incubation with all-*trans* retinal and 13-ethyl retinal in 50 mM phosphate buffer at pH 6.5. The bOp concentration is 1.0×10^−5^ M and the retinal was added to have a molar ratio 1∶1.5 bOp/retinal. The band at 380 nm (*) is related with the absorbance of the free retinal. The bands that appear in the region of 500–600 nm (**) correspond to the retinal that has reached the RBP of the corresponding bOp and formed a protonated SB.

In the present study, our approach consisted in removing the retinal molecule yielding the apoprotein form of bR (bOp). After retinal removal, we monitored the incorporation of the AT and 13E-RET to the WT-and T90A-bOp by UV-Vis spectroscopy (Figure 3). The reconstituted T90A shows a blue-shifted absorbance maximum compared to the reconstituted WT, indicative for a different pocket accommodation for the analogue in T90A. Likewise, the spectrum of T90A reconstituted with 13E-RET indicates a more native-like retinal environment compared to T90A reconstituted with RET (550 nm for T90A-13E-RET and 546 nm for T90A-AT; Figure 3 and Table 1). On the other hand, a delay/acceleration in the rate of retinal incorporation suggests unfavourable/favourable retinal-protein interactions in the RBP of the bOp. The incorporation of AT to the WT-bOp was taken as the reference. It showed a half-lifetime of 0.9 minutes, whereas the reconstitution with 13E-RET showed a reconstitution rate of about 9-fold longer (Table 1). The accessibility of the RBP was restricted for T90A-bOp when compared to WT-bOp, as indicated by its ∼11-fold longer half-lifetime for AT incorporation, or the ∼13-fold longer lifetime for 13E-RET incorporation (Table 1).

**Table 1:** Characterization of the reconstituted wt-bOp and T90A-bOp with AT retinal and the analogue 13E-RET.

	WT-AT	T90A-AT	WT-13E-RET	T90A-13E-RET
**λ_max_ pigment (nm)** a	568	546	558	550
**t_1/2_ reconstitution (min)** b	0.9±0.03	10.2±0.1	7.9±0.5	11.6±0.5
**Light-adaptation (nm)** c	558(DA)-568(LA)	Not observed	Not observed	Not observed
**t_1/2_ NH_2_OH (h)** d	6.1±0.003	5.1±0.02	4.1±0.001	3.7±0.02
**T^a^ denat. pH 7 (°C)** e	96.0±0.6	87.1±0.3	88.1±2.1	86.3±0.9
**pK_a_ H_2_O** f	4.6	6.1	4.7	6.7
**pK_a_ KCl 150 mM** g	3.9	5.8	4.5	6.4
**Transport** h	100%	∼10%	26%	∼<10%

a Maximum absorbance in the visible spectrum of the bound retinal.

b Half-life for the reconstitution of the bOps with all-trans and 13E-RET.

c Change in the absorbance maximum of the pigment band after incubation of the chromoproteins in the dark. DA, dark-adapted; LA, light-adapted.

d Half-life of hydrolysis of suspensions 10 µM of chromoprotein with 40 mM NH_2_OH in 5 mM phosphate buffer.

e Tm of thermal denaturation of the proteins measured as the retinal release with temperature.

f pKa of Asp85 in water.

g pKa of Asp85 in 150 mM KCl.

h Indirect measurement of the proton transport function in the chromoproteins measured as the light-induced transient absorbance change of a 15 µM protein suspension in the presence of 50 µM pyranine dye.

The DA absorbance maximum for WT-bOp reconstituted with AT after overnight incubation in darkness at room temperature was 558 nm, 10 nm blue shifted in respect to the LA form, in agreement with the observed value for WT bR [15]. This wavelength arises from the isomeric mixture of 13-*cis* (550 nm) and all-*trans* (568 nm) retinal, present in a 2∶1 ratio in the DA state [31]. After illumination (light adaptation), this maximum shifted to 568 nm as a result of the nearly complete conversion of the 13-*cis* to all-*trans*-retinal chromophore (Table 1).

The DA form of T90A-AT (one month in darkness) showed only a 2 nm shift in the absorbance maximum in respect to the LA form, and a slightly decreased extinction molar coefficient [20], suggesting defective light-adaptation. Indeed, the retinal isomeric composition in both the LA and DA states was approximately the same and similar to WT bR in the DA state as seen by HPLC (Figure S1 and Methods S1). This shows that mutation of Thr90 to Ala favors the 13-*cis*-retinal isomer and impairs the normal isomerization of the 13*-cis*-retinal to all-*trans* retinal by light. The same abnormal light adaptation behavior was observed for T90A-13E-RET, showing only a ∼2 nm shift upon a month in the dark. WT-13E-RET shows also this unusual behavior (Figure S2 and Figure S3) [32].

### Stability of Reconstituted Proteins

**Figure 4:**
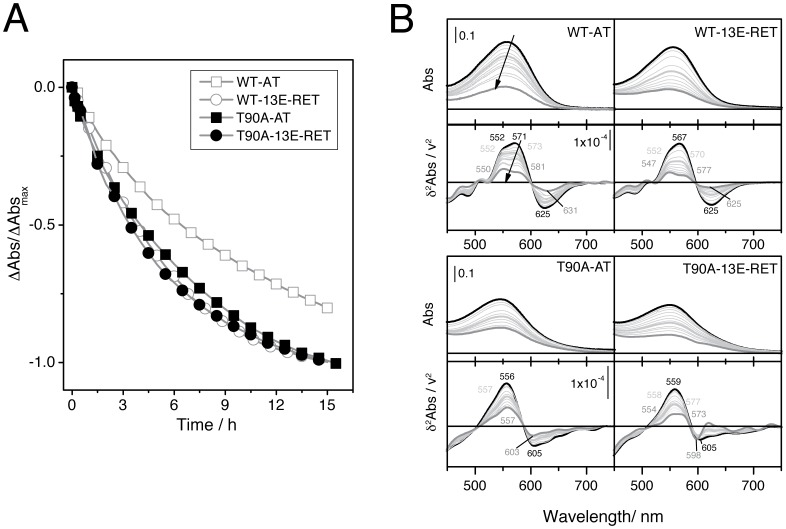
Chemical stability denaturation experiments. **A**. Kinetics of retinal hydrolysis of the four chromoproteins by hydroxylamine hydrochloride. The molar ratio protein/NH_2_OH was 1∶4000. The measurements were done in 5 mM phosphate buffer pH 7.5 in the dark. **B**. Absorbance spectra of the denaturation process (upper panels) compared to the minus second derivative of the absorbance spectra (lower panels) for each reconstituted protein (as indicated in the figure). For sake of clarity, initial, mid and final spectra of the reaction are plotted with thicker lines.

Chemical and thermal stabilities of the retinal-reconstituted bOps were monitored by UV-Vis spectroscopy. The reaction of hydroxylamine in the dark shows a half-lifetime of 6.1 hours for WT-AT compared to 5.1 hours for T90A-AT (Figure 4A and Table 1). The accessibility of hydroxylamine for the proteins reconstituted with the 13E-RET analogue was slightly increased since the hydrolysis occurred with a lower half-lifetime (4.1 and 3.7 hours for WT-13E-RET and T90A-13E-RET, respectively; Figure 4A and Table 1). Selected spectra for the chemical stability analysis are shown in Figure 4B. The minus second derivative is also represented to better characterize the hydrolysis kinetics, highlighting the differences between samples (lower panels in Figure 4B). For the sake of clarity, the spectral region between 250–450 nm is not displayed, because of the background absorbance of the retinal oxime and its low signal-to-noise ratio. The absorbance band of the pigment in the reconstituted WT-AT chromoprotein was composed of two main bands with a maximum at 552 and 571 nm. For WT-13E-RET both peaks were slightly blue-shifted, but for T90A-bOp reconstituted with AT or 13E-RET there was only one band at 556 and 559 nm, respectively. However, for T90A-13E-RET the two bands were present in the intermediate species that appear along the reaction, with a position in the spectrum similar to WT-AT.

**Figure 5:**
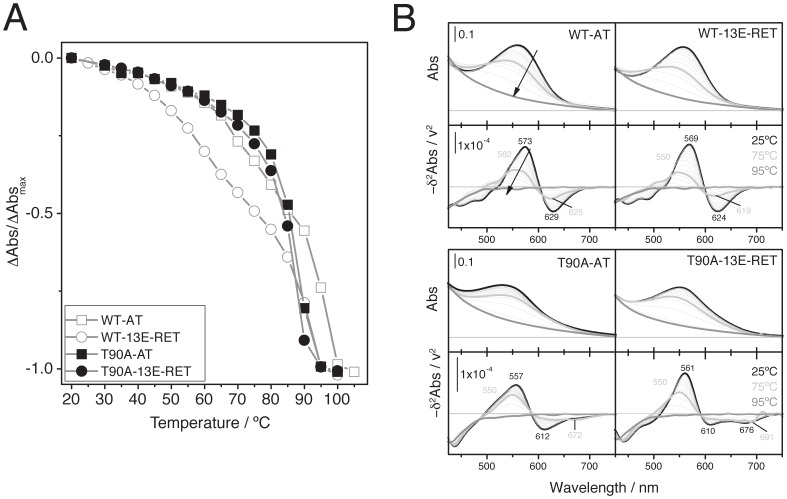
Thermal stability denaturation experiments. **A**. Kinetics of retinal release by thermal denaturation from suspensions of 10 µM wt-bOp and T90A-bOp reconstituted with AT and 13E-RET in 50 mM phosphate buffer. The chromoproteins were heated with an increment of 5°C and a stabilization time of 8 minutes before acquiring the absorbance spectrum. **B**. Absorbance spectra of the denaturation process (upper panels) compared to the minus second derivative of the absorbance spectra (lower panels) for each reconstituted protein (as indicated in the figure). For sake of clarity, initial, mid and final spectra of the reaction are plotted with thicker lines.

In the case of the RBP thermal stability, the retinal was released with a T_m_ of 96.0°C for WT-AT compared to 87.1°C for T90A-AT (Table1 and Fig. 5A). This decreased RBP stability for the T90A-AT agrees with the trend shown previously for T90A [19]. There was virtually no difference between T90A-AT and T90A-13E-AT (87.1 and 86.3°C, respectively; Table 1). Selected spectra for the thermal stability analysis are shown in Figure 5B. The minus second derivative (Figure 5B lower panels) showed a blue shift of the main bands for WT-13E-RET, T90A-AT and T90A-13E-RET compared to WT-AT, as described previously for the hydroxylamine reaction. However, the main peak is less blue-shifted in the T90A-bOp reconstituted with 13E-RET compared to T90A-AT (561 and 557 nm, respectively). The thermal transitions at the maximum absorbance peak are shown in Figure 5A.

Another main difference between the reconstituted WT and T90A-bOps is the region at 600–700 nm. This spectral region also reflects environmental changes in Asp85, the counterion of the protonated SB (further results on Asp85 below). Taking both chemical/thermal denaturation experiments into account, we can observe that for WT reconstituted proteins this region shows a single transition centered at 625 nm, the spectra for T90A reconstituted proteins show a more complex profile (Fig. 4B and 5B). The broader bandshape for T90A at the low energy range region (600–700 nm) argues for a distorted SB and Asp85 environments, even indicating partial protonation of the Asp85 (see Asp85 *pK_a_* results below).

### Asp85 Environment

The *pK_a_* of Asp85 is a sensitive parameter to probe the environment of the RBP, and specially the interaction of Asp85 with the retinal SB [33]. As shown in Table 1, the *pK_a_* values for WT-AT in deionised water and 150 mM KCl buffer were 4.6 and 3.9 respectively. For T90A-AT, these values rose to 6.1 and 5.8, respectively. The difference of about 1.5–2 pH units between WT and T90A, agrees with the difference observed for the WT and T90A bRs [19], indicating that the retinal environment in the vicinity of the SB is distorted for the T90A-AT, in a way that destabilizes the ionic form of Asp85.

In T90A-13E-RET, the pK_a_ of Asp85 was slightly affected by the 13E-RET-protein contacts when compared to T90A-AT, both in low and high ionic strength (Table 1). In view of that, one can say that the substitution of AT by 13E-RET in the T90A mutant did not recover the Asp85 native environment. Taking into account the previous results on chemical/thermal denaturation experiments (see above), it is evident that the incorporation of the 13E-RET to the T90A RBP affects directly the Asp85 and SB environments.

### Photocycle and Transport

**Figure 6:**
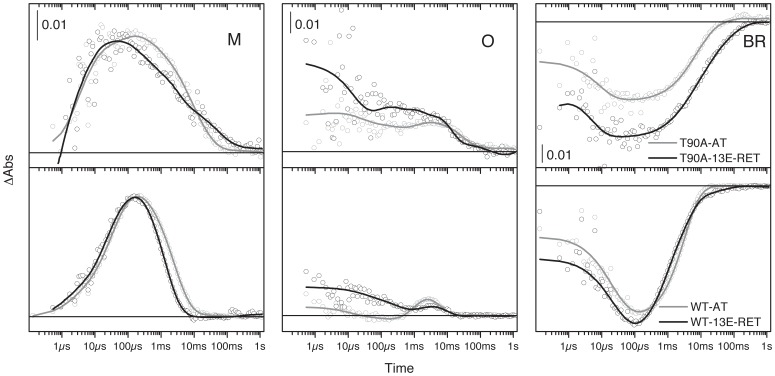
Photocycle characterization. Flash-induced transient absorbance change of a 15 µM protein suspension of the four chromoproteins at 410 nm (M), 660 nm (O) and 570 nm (BR). The samples were prepared in 3 mM phosphate buffer and 150 mM KCl at pH 7 and illuminated before starting the measurements to allow light adaptation. As indicated in the plot, upper panels: T90A-AT (grey circles and grey fitting line) and T90A-13E-RET (black circles and black fitting line); lower panels: WT-AT (grey circles and grey fitting line) and WT-13E-RET (black circles and black fitting line).

Flash-Photolysis UV-Vis spectroscopy was used to monitor the absorbance transient changes related to different photocycle intermediates. As shown in Fig. 6 (left panels), WT-13E-RET has a slightly faster M intermediate decay compared to WT-AT, in keeping with previous reports [34]. Both T90A-AT and T90A-13E-RET showed similar M intermediate formation kinetics, faster than WT. However, the decay showed at least two components for T90A-13E-RET, and although the first component forms faster (Fig. 6), the second has a longer lifespan (about 2-times) than T90A-AT. The O intermediate kinetics was distorted for both T90A-AT and T90A-13E-RET, compared to each other and compared to WT-AT (Fig. 5 mid panels). The abnormal high transient absorbance changes in the early time for T90A are likely to come from the contribution of the photocycle of the 13-*cis* retinal, which gives rise to a K-like intermediate relaxing to the ground-state without SB deprotonation, and thus without proton transport [35]. This K-like intermediate contains an all-*trans* retinal, and thus similar absorption maxima as the O intermediate of the normal all-*trans* photocycle. A delay in BR recovery was observed for reconstituted T90A compared to WT (Fig. 6 right panel), in accordance with the slower decay of the M intermediate. The maximum transient absorbance changes at 560 nm are lower for T90A-AT tan for WT-AT, pointing towards less bR molecules entering into the photocycle for the same laser excitation energy, and thus for a lower quantum yield for retinal photo-isomerization. Reconstitution with 13E-RET increases the transient absorbance changes for T90A at 560 nm, suggesting a recovery to WT levels of the quantum yield for the retinal photo-isomerization. Proton uptake and release in the bR photocycle were probed by the protonation changes of pyranine, monitored by pyranine transient absorbance changes in the visible. The magnitude of pyranine absorbance changes was measured, and used as an indirect indication of proton transport efficiency (Table 1), indicating lower transport efficiency (of about 10% of WT) for both reconstituted T90A proteins. WT-13E-RET showed slightly better transport than T90A reconstituted proteins, about 26% of WT (Table 1). Part of this decrease in transport efficiency can be accounted for by the presence in the LA form of T90A and WT-13E-RET of 13-*cis* retinal, whose photocycle is inactive for proton pumping. In addition, the fraction of molecules with protonated Asp85, larger for T90A than for WT due to the higher pKa of Asp85, will be impaired for proton transport (see *Asp85 environment* section). Additional contribution to the reduced transport efficiency can arise from reduced quantum efficiency for retinal photo-isomerization or reduced protein stability.

## Discussion

The architecture of a functional active site of a membrane protein is not only defined as the result of its close environment, but also by the overall topology in the membrane. The case of retinal proteins is not an exception. However, we can restrict the chromophore as the core of the protein, assuming the network of interactions established between the retinal and the protein as the initial and pivotal factor for the outwards propagation of the signal from the retinal to the rest of the protein and its arrangement in the membrane. Here, in an attempt to perform a phenotype recovery of the T90A mutant, we have used a retinal analogue in which the methyl group in the C_13_ hydrocarbon chain was substituted for an ethyl group (Figure 2). This extra group in position C_13_ of the retinal was conceived to facilitate the interaction with the alanine residue in the T90A mutant, mimicking the lost steric interaction between Thr90 and the C_13_-methyl moiety of the retinal molecule. Interestingly, our protein design attempt did not recover the WT phenotype, giving indirect evidence for the importance of the hydrogen bonding interactions established by Thr90 [19], [20].

In our previous studies and through several observations, it has been already shown that the T90A mutation distorted the excitation of the retinal in kept in the darkness, as well as the retinal isomeric composition in light-adapted conditions. In the present study, we show how the extra space for the accommodation of the chromophore in the RBP provided by the mutation favors the 13-*cis* retinal isomer (Figure S1), impairing the isomerization to all-*trans*, necessary for the proton transport [32]. In the WT the bulkier C_13_ group of the 13E-RET analogue may collide with the side-chains configuring the RBP, promoting an abnormal dark adaptation. This seems to indicate that the residues in close contact with the C_13_ group of the retinal molecule (Trp182, Leu93, Tyr185 and Thr90) are essential for the retinal isomerization and consequently a normal photocycle [9], [36]. In our study, distorted kinetics for O intermediate and BR recovery in WT and T90A bOps reconstituted with 13E-RET could be due to an altered interaction of the retinal analogue with Leu-93, and the Thr-90 in the case of the reconstituted WT-bOp. Leu-93 interacts with the 13-methyl group of retinal [37] in a similar manner that Thr-90. The bulkier ethyl group in the analogue could modify that interaction that seems to influence the reisomerization of the retinal during the photocycle at the N→O step. In the L93A mutant there is a long-living O intermediate and a slow N recovery [37], [38]. Toth-Boconadi et al. [38] postulated the presence of two O intermediates in the photocycle of L93A, one short-living with 13-*cis*-retinal configuration, and a late O intermediate with a twisted all-*trans*-retinal conformation that was detected also in WT bR cycle. The lost interaction between the terminal methyl groups of Leu-93 and the 13-methyl of retinal in L93A would hinder the retinal reisomerization. A bulkier group in the retinal analogue at C_13_ could explain those alterations observed in the photocycle of WT-13E-RET and T90A-13E-RET. Similarly, the loss of the interaction between the 13-methyl group of retinal and Thr-90 in T90A mutant would explain the distorted equilibrium of the retinal isomerization in the LA form (Figure S1) and the alteration in the photocycle observed in T90A-AT. Nevertheless, the L93A mutant does not show any decrease in the proton transport efficiency [37], as observed for the T90A mutant [20]. In contrast to the T90A mutant, the RBP of the T90V is slightly better suited to accommodate the all-*trans*-retinal, restoring an additional 10% the proton transport pumping of bR when compared to T90A [20]. This is not observed in the T90A-13E-RET, in which the proton transport remains around 10% of WT. This lack of accommodation of the retinal in T90A is also translated into the Asp85 behavior, where the constraints introduced in the RBP by the 13E-RET analogue facilitates the protonation of Asp85, shifting its pKa to higher values. This is also coherent with the faster appearance of the M intermediate in the photocycle of T90A (Fig. 5), where the SB proton is transferred to Asp85.

The active site functionality and stability depend on the presence of crucial hydrophilic residues embedded in the hydrophobic core of a transmembrane protein [39]. As shown here, the chemical and thermal stability of the RBP are compromised in WT by incorporating a bulkier chromophore that clashes with the Cγ_Thr90_, likely pointing the hydroxyl group of Thr90 in orientations that hinder the polar interactions with Asp115 and Trp86, recreating the behavior of the T90A mutant. However, the biphasic transition shown by the WT in the thermal release of both the AT and 13E-RET molecules, also indicates that the presence of the Cγ_Thr90_ is required for the proper accommodation of both retinal molecules. The incorporation of a bulkier chromophore in the RBP of the T90A accommodates slightly better the new chromophore by imitating the van der Waals interaction. Therefore, any change of volume, either increment or reduction, in the retinal vicinity affects the stability and functionality of the protein significantly [40], [41], [42]. This is reflected in the broad absorbance of the retinal of T90A in the chemical/thermal denaturation experiments, extending well until the 700 nm spectral region, suggesting heterogeneity in the retinal environment. The fact that the steric interaction of Ala90 with the retinal analogue is not enough to recover the functionality of the protein, is due most probably to the absence of the hydrogen bond network established by residue 90; that is, including a bulkier group at the retinal’s C_13_ in T90A eases slightly the accommodation of the analog in the retinal pocket, but has no beneficial effect on the function.

Recently, Joh et al. have determined the structures of the D115A and T90A/D115A mutants showing that the contribution to stability of the Thr90-Asp115 hydrogen bond is modest, although it corresponds to the strongest interactions among the studied hydrogen bonds [21]. However, the T90A mutant is so unstable that could not be crystallized (as we demonstrated earlier through a variety of biophysical techniques). This is an interesting observation, because it evidences the important alterations of Asp115 in the T90A mutant. From FTIR measurements, it was shown that unlike in WT Asp115 is deprotonated in the T90A mutant at neutral and even at slightly alkaline pH [20]. Therefore, in the WT the interaction of Asp115 with Thr90 controls the *pK_a_* of Asp115 and stabilizes its protonated form. In T90A, the presence of the negative charge of the deprotonated Asp115 embedded in a transmembrane hydrophobic core is likely to impair not only the stability (our experimental data [20] and Bowie’s Lab [21]), but also the dynamics of T90A (our experimental data [19], [20]). On the other hand, the expression yield of T90A mutant compared to WT shows a 10-fold decrease (data not shown) that may be representative of a restrictive folding in the membrane. The 2D-arrangement of bR may be sufficient to compensate partially this negative polar charge embedded in the membrane through lipid-protein interactions. Additionally, it is feasible that the water network is rearranged and extended to compensate this charge in some measure, disturbing the proton path and affecting the function. From a dynamics perspective (the photocycle), our previous results [19], [20], [22] agree with this view, showing that the Thr90-Asp115 hydrogen bond and the hydrogen bond network established in the hydrophobic core of bR, are essential for the propagation of the signal from the RBP to the rest of the protein. Our present results indirectly reinforce the importance of these hydrogen bonds, since restoring the steric interaction at the level of the C_13_ of retinal adds nothing to the function of the reconstituted protein.

## Supporting Information

Figure S1 **Retinal isomer composition analysis.** HPLC of Dark and light adapted (DA and LA) T90A (black lines) and WT (grey lines) showing there is not light adaptation for the mutant.
(TIF)

Figure S2 **Dark-light adaptation.** Dark-light adaptation of the four chromoproteins at 10 µM in 50 mM phosphate buffer at pH 6.5. The differences in scattering are due to the aggregation during the time of dark adaptation (more than one month).
(TIF)

Figure S3 **Dark-light adaptation.** Difference spectra between the light and the dark-adapted form of a 10 µM suspension of the four chromoproteins in 50 mM phosphate buffer. The suspensions have been kept in dark conditions for more than a month and a spectrum of each protein was acquired. Afterwards the suspension was illuminated for one minute at maximum intensity and another spectrum was recorded.
(TIF)

Methods S1 Methods for the retinal analog synthesis and the HPLC procedures.
(PDF)
