# Supplementary material for: Probing a Polar Cluster in the Retinal Binding Pocket of Bacteriorhodopsin by a Chemical Design Approach
Source: PLoS One. 2012 Aug 3;7(8):e42447. doi: 10.1371/journal.pone.0042447 (PMC3411786; doi:10.1371/journal.pone.0042447)
Supplement: Methods S1 — Methods for the retinal analog synthesis and the HPLC procedures. (PDF) [file pone.0042447.s004.pdf]

Probing a Polar Cluster in the Retinal Binding Pocket of Bacteriorhodopsin by a Chemical Design Approach

Rosana Simón-Vázquez[1], Marta Domínguez[2], Víctor A. Lórenz-Fonfría[1], Rosana Álvarez[2], José-Luís Bourdelande[3], Ángel R. de Lera[2], Esteve Padrós[1] and Alex Perálvarez-Marín[1,*].

[1] Centre d'Estudis Biofísics, Universitat Autònoma de Barcelona, 08193 Cerdanyola del Vallés, Barcelona, Spain
[2] Departamento de Química Orgánica. Facultad de Química. Universidade de Vigo, 36310 Vigo, Spain
[3] Departamento de Química (Química Orgánica) Universitat Autònoma de Barcelona, 08193 Cerdanyola del Vallés, Barcelona, Spain

SUPPORTING INFORMATION

Supporting Materials and Methods

HPLC Analysis.
HPLC analysis of the retinal isomeric state of WT and T90A was performed as described previously (ref), using DA (five weeks in darkness at 4 ^{o}C) and LA samples. First, the retinal was hydrolyzed and released at 4 ^{o}C by adding bR to an excess of methanol solution and 500 mM hydroxylamine. The formed retinal oximes were transferred to hexane, and loaded to a high-performance liquid chromatograph (HPLC) equipped with a silica column (6.0×150 mm; YMC-Pack SIL). The elution solvent (flowing at 1.0 mL/min) was composed of 12% (v/v) ethyl acetate and 0.12% (v/v), and the absorbance at 360 nm was recorded as a function of the retention time. Assignment of the peaks to retinal isomers was performed by comparing them with previously recorded patterns from retinal oximes of authentic all-*trans*- and 13-*cis*-retinals (Kawanabe *et al.* 2006). The whole process was performed under dim red light conditions.

Kawanabe A, Furutani Y, Jung KH, Kandori H. FTIR study of the photoisomerization processes in the 13-cis and all-trans forms of Anabaena sensory rhodopsin at 77 K. Biochemistry. 2006 Apr 11;45(14):4362-70.

Experimental Section for Synthesis
General. Solvents were dried according to published methods and distilled before use. HPLC grade solvents were used for the HPLC purification. All other reagents were commercial compounds of the highest purity available. All reactions were carried out under argon atmosphere, and those not involving aqueous reagents were carried out in oven-dried glassware. Analytical thin layer chromatography (TLC) was performed on aluminium plates with Merck Kieselgel 60F254 and visualised by UV irradiation (254 nm) or by staining with solution of phosphomolibdic acid. Flash column chromatography was carried out using Merck Kieselgel 60 (230-400 mesh) under pressure. High performance liquid chromatography was performed using a Waters instrument using a dualwave detector (254 and 300 nm) with a Preparative Nova Pak®

HR silica, 60 Å, 19 x 300 mm and 95:5 hexane/ethyl acetate as eluent. UV/Vis spectra were recorded on a Cary 100 Bio spectrophotometer using MeOH as solvent. Infrared spectra were obtained on JASCO FT-IR 4200 spectrophotometer, from a thin film deposited onto a NaCl glass. Specific rotation was obtained on JASCO P-1020. Mass spectra were obtained on a Hewlett-Packard HP59970 instrument operating at 70 eV by electron ionisation. High Resolution mass spectra were taken on a VG Autospec instrument. ^{1}H NMR spectra were recorded in $CDCl_3$, C_6D_6 and $(CD_3)_2CO$ at ambient temperature on a Bruker AMX-400 spectrometer at 400 MHz with residual protic solvent as the internal reference ($CDCl_3$, δ_H = 7.26 ppm; C_6D_6, δ_H = 7.16 ppm; $(CD_3)_2CO$, δ_H = 2.05 ppm); chemical shifts (δ) are given in parts per million (ppm), and coupling constants (*J*) are given in Hertz (Hz). The proton spectra are reported as follows: δ (multiplicity, coupling constant *J*, number of protons, assignment). ^{13}C NMR spectra were recorded in $CDCl_3$, C_6D_6 and $(CD_3)_2CO$ at ambient temperature on the same spectrometer at 100 MHz, with the central peak of $CDCl_3$ (δ_C = 77.0 ppm), C_6D_6 (δ_C = 128.0 ppm) or $(CD_3)_2CO$ (δ_C = 30.8 ppm) as the internal reference. DEPT135 are used to aid in the assignment of signals in the ^{13}C NMR spectra.

(2E,4E,6E)-8-(tert-Butyldiphenylsilanyloxy)-6-ethyl-2-methyl-octa-2,4,6-trien-1-ol 7. To a cooled (0 °C) solution of (2*E*,4*E*,6*E*)-6-bromo-8-*tert*-butyldiphenylsilanyloxy)-2-methyl-octa-2,4,6-trien-1-ol **2** (0.116 g, 0.25 mmol) and $NiCl_2$dppp (0.014 g, 0.025 mmol) in THF (2.5 mL) in a sealed tube, EtMgBr (0.741 mL, 1M in THF, 0.741 mmol) was added. After stirring at 40 °C for 4h, EtOH was added (3 mL) and the mixture was filtered and concentrated. The residue was purified by column chromatography (silica gel, 95:5 hexane/ethyl acetate) to afford 0.068 g of a yellow oil (66%) which was identified as (2*E*,4*E*,6*E*)-8-(*tert*-butyldiphenylsilanyloxy)-6-ethyl-2-methyl-octa-2,4,6-trien-1-ol **7**. **^{1}H-NMR** (400.16 MHz, C_6D_6): δ 7.9-7.8 (m, 4H, ArH), 7.3-7.2 (m, 6H, ArH), 6.54 (dd, *J*= 15.3, 10.9 Hz, 1H, H_4), 6.34 (d, *J* = 15.3 Hz, 1H, H_5), 5.96 (d, *J*= 10.7 Hz, 1H, H_3), 5.73 (t, *J*= 6.2 Hz, 1H, H_7), 4.56 (d, *J*= 6.4 Hz, 2H, $2H_8$), 3.69 (d, *J* = 3.7 Hz, 2H, $2H_1$), 2.20 (q, *J*= 7.4 Hz, 2H, C_6-$\underline{CH_2}CH_3$), 1.58 (s, 3H, C_2-CH_3), 1.19 (s, 9H, $Si(CH_3)_3$), 1.05 (t, *J*= 7.4 Hz, 3H, C_6-$CH_2\underline{CH_3}$) ppm. **^{13}C-NMR** (100.62 MHz, $CDCl_3$): δ 139.8 (s), 137.4 (s), 135.5 (d, 4x), 133.8 (s, 2x), 129.6 (d, 2x), 128.6 (d), 127.7 (d), 127.5 (d, 4x), 125.6 (d), 124.6 (d), 68.4 (t), 60.4 (t), 26.7 (q, 3x), 26.2 (t), 19.1 (s), 14.2 (q), 13.2 (q) ppm. **MS** (FAB^+): *m/z* (%) 421 ($[M+1]^+$, 6), 420 (M^+, 13), 419 (12), 404 (10), 403 (29), 307 (15), 289 (11), 239 (11), 235 (10), 200 (18), 199 (100), 198 (12), 197 (51). **HRMS** (FAB^+): Calcd. for $C_{27}H_{36}O_2Si$ ($[M]^+$) 420.2485; found, 420.2484. **IR** (NaCl)**:** ν 3500-3100 (br, O-H), 2960 (s, C-H), 2929 (s, C-H), 2856 (m, C-H), 1670, 1470, 1110 cm^{-1}. **UV** (MeOH): λ_{max} 280 nm.

(2E,4E,6E)-8-(tert-Butyldiphenylsilanyloxy)-6-ethyl-2-methylocta-2,4,6-trien-1-al 4. To a solution of (2*E*, 4*E*, 6*E*)-8-(*tert*-butyldiphenylsilanyloxy)-6-ethyl-2-methyl-octa-2,4,6-trien-1-ol **7** (0.101 g, 0.24 mmol) in CH_2Cl_2 (6 mL) was added MnO_2 (0.38 g, 4.32 mmol) and the suspension was stirred for 3h. The mixture was filtered throught Celite and the solvents were removed. The residue was purified by column chromatography (silica gel, 95:5 hexane/ ethyl acetate) to afford 0.079 g of a yellow oil (79%) that was identified as (2*E*,4*E*,6*E*)-8-(*tert*-butyldiphenylsilanyloxy)-6-ethyl-2-methyl-octa-2,4,6-trien-1-ol **4**. **^{1}H-NMR** (400.13 MHz, C_6D_6): δ 9.37 (s, 1H, H_1), 7.9-7.8 (m, 4H, ArH), 7.3-7.2 (m, 6H, ArH), 6.4-6.3 (m, 2H, $H_4 + H_5$), 6.2-6.1 (m, 1H, H_3), 5.82 (t, *J*= 6.4 Hz, 1H, H_7), 4.48 (d, *J*= 6.4 Hz, 2H, $2H_8$), 2.02 (q, *J* = 7.4 Hz, 2H, C_6-$\underline{CH_2}CH_3$), 1.73 (d, *J* = 1.1 Hz, 3H, C_2-CH_3), 1.19 (s, 9H, $Si(CH_3)_3$), 0.94 (t, *J*= 7.4 Hz, 3H, C_6-$CH_2\underline{CH_3}$) ppm. **^{13}C-NMR** (100.62 MHz, $(CD_3)_2CO$): δ 194.8 (d), 149.6 (d), 140.0 (s), 138.6 (s),

137.7 (s), 136.5 (d, 4x), 134.6 (s, 2x), 133.4 (d), 130.9 (d, 2x), 128.9 (d, 4x), 125.8 (d), 61.3 (t), 27.4 (q, 3x), 26.8 (t), 19.9 (s), 13.8 (q), 9.7 (q) ppm. **MS** (FAB^+): *m/z* (%) 419 ($[M+1]^+$, 18), 418 (M^+, 15), 417 (14), 361 (21), 239 (13), 221 (12), 200 (18), 199 (96), 198 (12), 197 (51), 183 (15), 165 (16), 164 (20), 163 (100). **HRMS** (FAB^+): Calcd. for $C_{27}H_{35}O_2Si$ [$(M+1)^+$], 419.2406; found, 419.2413. **IR** (NaCl)**:** ν 2961 (s, C-H), 2930 (s, C-H), 2857 (m, C-H), 1675 (s, C=O), 1612, 1111 cm^{-1}. **UV** (MeOH): λ_{max} 319 nm.

(2E,4E,6E,8E)-tert-Butyl-[3-ethyl-7-methyl-9-(2,6,6-trimethylcyclohex-1-en-1-yl)nona-2,4,6,8-tetraen-1-yl]diphenylsilane 5. A cooled (-30 ºC) emulsion of triphenyl-(2,6,6-trimethylcyclohex-1-enylmethyl)phosphonium bromide **3** (0.182 g, 0.38 mmol) in THF (4 mL) was treated with *n*-BuLi (0.270 mL, 1.41 M in hexanes, 0.38 mmol) and stirred for 30 min. The mixture was cooled down to -78 °C and a solution of (2*E*,4*E*,6*E*)-8-(*tert*-butyldiphenylsilanyloxy)-6-ethyl-2-methyl-octa-2,4,6-trien-1-ol **4** (0.133 g, 0.32 mmol) in THF (4 mL) was added. The resulting mixture was allowed to warm to 25 °C for 14 h, and H_2O (8 mL) was added. The reaction was extracted with Et_2O (3x) and the organic layers were washed with brine (3x), dried (Na_2SO_4) and the solvent was evaporated. The residue was purified by column chromatography (silica gel, hexane) to afford 0.12 g (70%) of a yellow oil identified as (2*E*,4*E*,6*E*,8*E*)-*tert*-butyl-[3-ethyl-7-methyl-9-(2,6,6-trimethylcyclohex-1-en-1-yl)nona-2,4,6,8-tetraen-1-yl]diphenylsilane **5** and 0.024 g (16%) of another yellow oil identified as (6*E*,8*E*,10*E*)-12-(*tert*-butyldiphenylsilanyl)-10-ethyl-6-methyl-dodeca-6,8,10-trien-5-ol **6**. Data for (2*E*,4*E*,6*E*,8*E*)-*tert*-butyl-[3-ethyl-7-methyl-9-(2,6,6-trimethylcyclohex-1-en-1-yl)nona-2,4,6,8-tetraen-1-yl]diphenylsilane **5**: **^{1}H-NMR** (400.16 MHz, C_6D_6): δ 7.9-7.8 (m, 4H, ArH), 7.2-7.1 (m, 6H, ArH), 6.71 (dd, *J*= 15.2, 11.2 Hz, 1H, H_{11}), 6.35 (d, *J*= 15.2 Hz, 1H, H_{12}), 6.28 (m, 2H, $H_7 + H_8$), 6.04 (d, *J*= 11.2 Hz, 2H, H_{10}), 5.75 (t, *J*= 6.4 Hz, 1H, H_{14}), 4.57 (d, *J*= 6.4 Hz, 2H, $2H_{15}$), 2.21 (q, *J*= 7.5 Hz, 2H, C_{13}-$\underline{CH_2}CH_3$), 2.0-1.9 (m, 2H, $2H_4$), 1.84 (s, 3H, C_9-CH_3), 1.80 (s, 3H, C_5-CH_3), 1.6-1.5 (m, 2H, $2H_3$), 1.5-1.4 (m, 2H, $2H_2$), 1.20 (s, 9H, $Si(CH_3)_3$), 1.13 (s, 6H, C_1-$(CH_3)_2$), 1.06 (t, *J*= 7.4 Hz, 3H, C_{13}-$CH_2\underline{CH_3}$) ppm. **^{13}C-NMR** (100.62 MHz, $(CD_3)_2CO$): δ 139.5 (s), 137.7 (d), 137.6 (s), 135.7 (s, 2x), 135.3 (d, 4x), 133.6 (s), 130.8 (d), 129.6 (d, 2x), 128.7 (s), 128.2 (d), 127.6 (d, 4x), 127.4 (d), 126.4 (d), 125.9 (d), 60.2 (t), 39.3 (t), 33.8 (s), 32.6 (t), 29.7 (q), 28.3 (q), 26.2 (q, 3x), 26.0 (t), 20.9 (q), 18.9 (t), 18.7 (s), 12.9 (q), 11.7 (q) ppm. **MS** (FAB^+): *m/z* (%) 539 ($[M+1]^+$, 30), 538 ($[M]^+$, 62), 537 (10), 481 (10), 348 (21), 283 (41), 281 (10), 269 (25), 239 (11), 213 (10), 201 (12), 200 (19), 199 (100), 198 (19), 197 (88), 195 (10). **HRMS** (FAB^+): Calcd. for $C_{12}H_{51}OSi$ ($[M+1]^+$), 539.3709; found, 539.3702. **IR** (NaCl)**:** ν 3360, 2924 (s, C-H), 2854 (s, C-H), 1654, 1633 cm^{-1}. **UV** (MeOH): λ_{max} 329nm. Data for (6*E*,8*E*,10*E*)-12-(*tert*-butyldiphenylsilanyl)-10-ethyl-6-methyl-dodeca-6,8,10-trien-5-ol **6**: **^{1}H-NMR** (400.13 MHz, $CDCl_3$): δ 7.7-7.6 (m, 4H, ArH), 7.4-7.3 (m, 6H, ArH), 6.42 (dd, *J*= 15.3, 10.8 Hz, 1H, H_8), 6.20 (d, *J*= 15.3 Hz, 1H, H_9), 5.96 (d, *J*= 10.8 Hz, 2H, H_7), 5.54 (t, *J*= 6.3 Hz, 1H, H_{11}), 4.39 (d, *J*= 6.4 Hz, 2H, $2H_{12}$), 4.02 (t, *J*= 6.6 Hz, 1H, H_5), 2.23 (q, *J*= 7.4 Hz, 2H, C_{10}-$\underline{CH_2}CH_3$), 1.75 (s, 3H, C_6-CH_3), 1.6-1.5 (m, 2H, $2H_4$), 1.4-1.3 (m, 2H, $2H_3$), 1.3-1.2 (m, 2H, $2H_2$), 1.07 (t, *J*= 7.4 Hz, 3H, C_{10}-$CH_2\underline{CH_3}$), 1.05 (s, 9H, $Si(CH_3)_3$), 0.90 (t, *J*= 7.0 Hz, 3H, H_1) ppm. **^{13}C-NMR** (100.62 MHz, $CDCl_3$): δ 140.2 (s), 139.0 (s), 135.6 (d, 4x), 133.9 (s, 2x), 132.5 (d, 2x), 128.7 (d), 127.8 (d), 127.6 (d, 4x), 126.2 (d), 124.7 (d), 77.7 (d), 60.5 (t), 34.7 (t), 27.8 (t), 26.8 (q, 3x), 26.3 (t), 22.6 (t), 19.2 (s), 14.0 (q), 13.29 (q), 12.1 (q) ppm. **MS** (FAB^+): *m/z* (%) 477 ($[M+1]^+$, 11), 476 ($[M]^+$, 11), 475 (11), 460 (17), 459 (42), 419 (18), 267 (17), 239 (10), 221 (13), 204 (15), 203 (66), 199 (100), 198 (13), 197 (57). **HRMS** (FAB^+): Calcd. for $C_{31}H_{44}O_2Si$, ($[M]^+$), 476.3111; found, 476.3120.

IR (NaCl)**:** ν 3450-3100 (br, O-H), 2959 (s, C-H), 2931 (s, C- H), 2858 (m, C-H), 1428, 1111 cm^{-1}. **UV** (MeOH): λ_{max} 282 nm

13-Ethylretinal 1. A cooled (0 °C) solution of (2*E*,4*E*,6*E*,8*E*)-*tert*-butyl-[3-ethyl-7-methyl-9-(2,6,6-trimethylcyclohex-1-en-1-yl)nona-2,4,6,8-tetraen-1-yl]diphenylsilane **5** (26.9 mg, 0.05 mmol) in THF (0.45 mL) was treated with *n*-Bu_4NF (0.075 mL, 1 M in THF, 0.075 mmol) and stirred for 2 h. The mixture was diluted with Et_2O (1 mL) and washed with an aqueous solution of $NaHCO_3$ (1x). The aqueous layer was extracted with Et_2O (3x) and the combinated organic layers were washed with brine (3x), dried (Na_2SO_4) and concentrated. The residue was used without further purification. To a solution of this compound in CH_2Cl_2 (1 mL) was added MnO_2 (78 mg, 0.9 mmol) and Na_2CO_3 (95 mg, 0.9 mmol), and the suspension was stirred at room temperature for 3 h. The mixture was filtered through Celite and the solvent was removed. The residue was purified by column chromatography (silica gel, 95:5 hexane/ethyl acetate) to afford 9.1 mg (60%, both steps) of a yellow oil identified as a mixture of (13*E*)-13-ethylretinal **1** and (13*Z*)-13-ethylretinal (13*Z*)-**1** in a 2:1 13*E*/13*Z* ratio which were separated by HPLC. Data for (13*E*)-13-ethylretinal **1**: **^{1}H-NMR** (400.13 MHz, C_6D_6): δ 10.04 (d, *J*= 7.7 Hz, 1H, H_{15}), 6.93 (dd, *J*= 15.2, 11.4 Hz, 1H, H_{11}), 6.35 (d, *J*= 16.2 Hz, 1H, H_7), 6.26 (d, *J*= 16.2 Hz, 1H, H_8), 6.03 (d, *J*= 11.4 Hz, 1H, H_{10}), 6.0-5.9 (m, 2H, H_{12} + H_{14}), 2.35 (q, *J*= 7.6 Hz, 2H, C_{13}-$\underline{CH_2}CH_3$), 2.0-1.9 (m, 2H, $2H_4$), 1.77 (s, 3H, C_9-CH_3), 1.76 (s, 3H, C_5-CH_3), 1.6-1.5 (m, 2H, $2H_3$), 1.5-1.4 (m, 2H, $2H_2$), 1.12 (s, 6H, C_1-$(CH_3)_2$), 0.91 (t, *J*= 7.4 Hz, 3H, C_6-$CH_2\underline{CH_3}$) ppm. **^{13}C-NMR** (100.62 MHz, $(CD_3)_2CO$): δ 190.0 (d), 160.7 (s), 140.4 (s), 137.5 (s), 137.3 (d), 133.3 (d), 131.9 (d), 129.9 (d), 129.6 (s), 128.8 (d), 127.6 (d), 39.3 (t), 33.9 (s), 32.6 (t), 28.2 (q, 2x), 20.9 (q), 19.8 (t), 18.8 (t), 15.1 (q), 12.0 (q) ppm. **MS** (EI^+): *m/z* (%) 298 ($[M]^+$, 22), 236 (14), 173 (13), 137 (18), 136 (12), 135 (12), 123 (14), 121 (18), 119 (15), 111 (14), 109 (18), 107 (13), 105 (11), 98 (12), 97 (27), 96 (15), 95 (30), 93 (14), 91 (11), 85 (17), 83 (33), 82 (20), 81 (57), 72 (19), 71 (29), 70 (16), 69 (100). **HRMS** (EI^+): Calcd. para $C_{21}H_{30}O$ ($[M]^+$), 298.2297; found, 298.2293. **IR** (NaCl)**:** ν 2961 (s, C-H), 2927 (s, C-H), 2863 (m, C-H), 1660 (s, C=O), 1578, 1116 cm^{-1}. **UV** (MeOH): λ_{max} 383 (22700) nm. Data for (13*Z*)-13-ethylretinal (13*Z*)-**1**: **^{1}H-NMR** (400.13 MHz, C_6D_6): δ 10.19 (d, *J*= 7.5 Hz, 1H, H_{15}), 6.96 (d, *J*= 15.0 Hz, 1H, H_{12}), 6.81 (dd, *J*= 15.0, 11.2 Hz, 1H, H_{11}), 6.37 (d, *J*= 16.2 Hz, 1H, H_7), 6.29 (d, *J*= 16.1 Hz, 1H, H_8), 6.06 (d, *J*= 11.2 Hz, 1H, H_{10}), 5.83 (d, *J*= 7.6 Hz, 1H, H_{14}), 2.00 (q, *J*= 7.4 Hz, 2H, C_{13}-$\underline{CH_2}CH_3$), 2.0-1.9 (m, 2H, $2H_4$), 1.79 (s, 3H, C_9-CH_3), 1.78 (s, 3H, C_5-CH_3), 1.6-1.5 (m, 2H, $2H_3$), 1.5-1.4 (m, 2H, $2H_2$), 1.33 (s, 6H, C_1-$(CH_3)_2$), 0.84 (t, *J* = 7.4 Hz, 3H, C_{13}-$CH_2\underline{CH_3}$) ppm. **^{1}H-NMR** (400.13 MHz, $(CD_3)_2CO$): δ 10.27 (d, *J*= 7.7 Hz, 1H, H_{15}), 7.42 (d, *J*= 15.1 Hz, 1H, H_{12}), 7.23 (dd, *J* = 15.1, 11.3 Hz, 1H, H_{11}), 6.40 (d, *J*= 15.9 Hz, 1H, H_7), 6.35 (d, *J*= 11.4 Hz, 1H, H_{10}), 6.24 (d, *J*= 16.1 Hz, 1H, H_8), 5.81 (d, *J*= 7.8 Hz, 1H, H_{14}), 2.57 (q, *J*= 7.5 Hz, 2H, C_{13}-$\underline{CH_2}CH_3$), 2.07 (s, 3H, C_9-CH_3), 2.1-2.0 (m, 2H, $2H_4$), 1.74 (s, 3H, C_5-CH_3), 1.7-1.6 (m, 2H, $2H_3$), 1.6-1.5 (m, 2H, $2H_2$), 1.19 (t, *J*= 7.5 Hz, 3H, C_{13}-$CH_2\underline{CH_3}$), 1.06 (s, 6H, C_1-$(CH_3)_2$) ppm. **^{13}C-NMR** (100.62 MHz, $(CD_3)_2CO$): δ 189.5 (d), 159.5 (s), 140.3 (s), 137.5 (s), 137.3 (d), 132.5 (d), 130.0 (d), 129.5 (s), 128.7 (d), 126.0 (d), 125.9 (d), 39.3 (t), 33.8 (s), 32.6 (t), 28.2 (q, 2x), 26.5 (t), 20.9 (q, C_5-CH_3), 18.8 (t), 12.9 (q), 11.9 (q) ppm. **MS** (EI^+): *m/z* (%) 299 ($[M+1]^+$, 23), 298 ($[M]^+$, 100), 283 (13), 269 (13), 173 (29), 159 (11), 145 (11), 133 (13), 131 (11), 119 (20), 109 (13), 105 (18), 95 (14), 91 (19). **HRMS** (EI^+): Calcd. For $C_{21}H_{30}O$, 298.2297 ($[M]^+$); found, 298.2294. **IR** (NaCl)**:** ν 2960 (s, C-H), 2927 (s, C-H), 2863 (m, C-H), 1661 (s, C=O), 1578, 1117 cm^{-1}. **UV** (MeOH): λ_{max} 377 (ε = 18500), 259 nm.
